# Linking STRs/SNPs and DNA methylation using massively parallel sequencing for potential forensic applications

**DOI:** 10.1007/s00414-025-03602-2

**Published:** 2025-10-21

**Authors:** Laura Schmelzer, Jerry Hoogenboom, Jana Naue

**Affiliations:** 1 Institute of Forensic Medicine, Medical Center – University of Freiburg, Medical Faculty – University of Freiburg, Freiburg, Germany; 2 Division of Biological Traces, Netherlands Forensic Institute, The Hague, The Netherlands

**Keywords:** DNA methylation, STR analysis, FDSTools, Massively parallel sequencing

## Abstract

DNA methylation is an important target for crime trace analysis. Amongst others, the combined analysis of tissue-specific differentially methylated DNA positions (tDMP) and person-identifying single nucleotide polymorphisms (iSNP) has demonstrated potential to link known mixture contributors to their respective body fluid. As STRs are the gold standard for individualization, we aimed to implement a pipeline to perform STR analysis of bisulfite converted DNA and to characterize DNA methylation sites in their close proximity, potentially identifying body fluid-varying methylation. We analyzed forensically used STR regions in blood, semen, saliva, buccal mucosa, nasal secretion, menstrual blood and vaginal fluid using amplicon-based bisulfite massively parallel sequencing. Furthermore, we evaluated the DNA methylation of eight known tDMPs for blood, semen, saliva and vaginal fluid and ten surrounding iSNPs. Successful STR analysis and allele structure reporting from bisulfite converted DNA was possible for 18 of 22 STRs. We identified D1S1677, FGA, D5S2800, SE33, D8S1179 and D19S433 flanking sites with varying DNA methylation between body fluids, but not sufficient discrimination power for use as exclusive body fluid identification markers. A first exemplary experiment showed STR potential for assignment of the corresponding contributor in mixtures, most promising for specific mixture cases including semen and second pre-determined body fluid. We confirmed known tDMPs and linked iSNPs as valuable targets for contributor assignment in mixtures. FDSTools was used with a newly generated library file for the naming of STRs in bisulfite converted DNA and allele-linked DNAm analysis usable and editable even for other research questions and applications.

**Supplementary Information:**

The online version contains supplementary material available at 10.1007/s00414-025-03602-2.

## Introduction

Short tandem repeat (STR) analysis by capillary electrophoresis (CE) is the gold standard for person identification when analyzing trace material in forensic investigations. In recent years, STR analysis using massively parallel sequencing (MPS) has become increasingly attractive. Compared to CE analysis, MPS offers enhanced multiplexing capacity and enables the analysis of the flanking region and STR allele sequence itself. This facilitates identification of additional variants and isoalleles, increasing the discriminative power for several STRs [1]. Recommendations by the ISFG on nomenclature as well as packages as STRNaming allow uniform reporting of allele structures [2, 3]. In addition to STRs, single nucleotide polymorphisms (SNPs) with high variability are useful for person identification (so-called identity SNPs, iSNPs), especially when DNA is highly degraded. Since most iSNPs are bi- or tri-allelic, they harbor a lower discriminative power than STRs, which can be compensated by increasing the number of analyzed iSNPs [4].

In addition to identifying the contributor(s) in a trace, determining the type of biological source is an important task. The presence of certain body fluids or tissues that may be specific to (parts of) organs can offer valuable insight into the circumstances of a crime. Currently, enzymatic and immunologic methods are widely used for this purpose, although several presumptive tests have suboptimal specificity, sensitivity and are each designed for only one body fluid [5]. Additionally, RNA and DNA methylation (DNAm) analysis are used for body fluid determination, offering the opportunity for simultaneous detection of relevant body fluids in a single reaction [6].

DNAm is a robust epigenetic mark that occurs predominantly at the C-5 position of cytosine followed by a guanine base (CpG) and plays an important role in transcriptional regulation, contributing to cell type-specific morphology and function [7]. Tissue-specific DNAm positions (tDMPs) have been identified by genome-wide DNAm profiling and validated to distinguish among body fluids, including blood, saliva, semen, vaginal secretion and menstrual blood [8–11]. These have been combined in diverse assays based on methylation-sensitive restriction enzymes or bisulfite conversion (e.g., SNaPshot, pyrosequencing, melt-curve analysis, quantitative PCR, MPS) for parallel body fluid analysis [12]. The use of DNAm as an analytical marker benefits from the robustness of DNAm upon environmental exposure and the DNAm analysis offers compatibility with routine DNA extraction procedures [10, 13, 14]. When performing DNAm analysis on low-template casework samples, it is important to consider that stochastic effects and additional DNA loss upon bisulfite conversion may affect DNAm measurements [15, 16].

Performing distinct STR/iSNP and DNAm/RNA analysis, the body fluid identification at best can be linked merely indirectly with the donors of the obtained STR profile. While earlier approaches have combined analysis of genetic and epigenetic markers into a single assay, they used targets located on widely separated DNA sites [17, 18]. Furthermore, the presence of trace mixtures is common and contributor deconvolution remains a challenging task. A highly relevant question pertains to the biological source type and contributor assignment in cases where the mixture consists of different body fluids, and when assignment cannot be done based on sex specificity e.g., semen in two-person male-female mixtures.

In 2016, Watanabe et al. published the first study on combined DNAm and neighboring iSNP analysis (here referred to as tDMP-iSNP loci) to assign contributors to their corresponding body fluid in mixtures [13]. They successfully assigned the contributors by allele-specific blood identification from mixed body fluid DNA, using bisulfite sequencing of allele-specific clones. This method is comparable to the idea of iSNP analysis in RNA transcripts for body fluid assignment [19, 20]. To date, several research groups have evaluated the combination of tDMP and iSNP sites using DNAm-sensitive PCR combined with pyrosequencing, SNaPshot, or using bisulfite-conversion MPS, respectively [21–23]. The MPS approach offers a direct link, as information of analyzed DNAm level and iSNP allele are on the same sequenced DNA molecule. The publications referenced above have focused on the analysis of semen, blood, and saliva. There is currently a lack of combined tDMP and iSNP markers for detection of vaginal secretion and the independent evaluation of tDMP-iSNP loci already identified. The use of iSNPs bears the major risk that known trace contributors in mixtures cannot be differentiated by a single iSNP in the case both contributors have the same genotype. Recently, semen-specific DNAm sites with neighboring nucleotide insertion or deletion (InDel) or low discriminative STRs [24] as well as blood- and semen-specific DNAm sites with neighboring microhaplotypes [25–27] have been reported. Holding up to six alleles per marker, the InDel/STR and microhaplotype systems have increased discriminative power and have successfully assigned semen and blood donors in mixture testing [24–26].

To the best of our knowledge, highly variable STRs commonly used in forensics have not been analyzed using bisulfite-based MPS. We intended to investigate the success of a reliable analysis of these complex structures especially after bisulfite conversion and to characterize their neighboring CpG sites. In this study, we considered 35 STRs resulting in the analysis of 18 STRs and the DNAm levels of 38 STR-flanking CpG sites in forensically relevant body fluids. Tissue-specific DNAm levels with strong differences between tissues would be needed for immediate body fluid determination, whereas slight differences and overlapping DNAm levels might still help to assign separately determined body fluids to known mixture contributors based on STR profiles. The analysis of STR and tDMP would have the advantage of higher discriminatory power compared to iSNPs and the alleles of the individuals may already be known from previous CE-based STR typing. For STR, iSNP and DNAm analysis we used FDSTools and an adapted library file for bisulfite-converted DNA. The development of this adapted tool will provide researchers with freely available software for other research questions and applications regarding allele-linked DNAm as well as more general STR analysis of bisulfite converted DNA, including ISFG-concordant allele structure reporting [2, 3].

Furthermore, this study aimed to evaluate eight tDMPs and nearby iSNPs, to counteract the risk that STR neighboring DNAm sites alone might show insufficient or no body fluid distinct DNAm levels. In addition, tDMP-SNP sites are suitable in degraded DNA samples. The combined analysis could therefore increase the success rate and discriminatory power in contributor assignment in pre-identified body fluids in mixtures. We included both known tDMP-iSNP loci [13, 21, 23] as well as published tDMPs with nearby iSNPs [9, 10, 28], as correct allele calling, tissue specificity and knowledge of cross-reactivity are essential.

## Material and methods

### Selection of STRs and iSNP sites included for consideration

35 autosomal STRs from the following commercial MPS kits were considered for the study: ForenSeq MainstAY/Signature (Qiagen, formerly Verogen, Hilden, Germany), Precision ID GlobalFiler NGS STR Panel v2 (Thermo Fisher Scientific (TFS), Waltham, MA, USA), IDseek OmniSTR Global (NimaGen, Nijmegen, The Netherlands) and PowerSeq 46GY System (Promega, Madison, WI, USA). The STRs were filtered according to the criteria that STRs must contain at least one CpG site within their repeat structure or at a maximum distance of ±100 bp from the STR repeat region (Table 1a+b).

**Table 1:** Selection of STRs with neighboring CpG sites for bisulfite-conversion MPS

						SNP affecting DNAm site	
	STR	location (GRCh38)	MPS kit	minimum distance to CpG [bp]	number of CpGs in PCR amplicon	CpG site	NCBI dbSNP ID, alleles, global MAF*
a	D21S11	chr21:19181973-19182101	NPTV	0	1		
	D1S1677	chr1:163590026-163590093	T	0	2		
	D7S820	chr7:84160205-84160276	NPTV	1	2	D7S820+10	rs16887642, G>A, 0.07
	PentaD	chr21:43636196-43636274	NPTV	1	2		
	D8S1179	chr8:124894863-124894914	NPTV	2	2		
	D14S1434	chr14:94842054-94842105	T	3	1	D14S1434-2	rs112858516, G>A, 0.01
	D3S4529	chr3:85803483-85803534	T	9	1		
	D20S482	chr20:4525692-4525747	NV	12	2	D20S482-12	rs77560248, C>T, 0.07
	TPOX	chr2:1489651-1489682	NPTV	17	6		
	D12ATA63	chr12:107928588-107928626	T	18	1		
	D5S2800	chr5:59403131-59403190	T	21	2	D5S2800+21	rs12187142, C>T, 0.13
	D2S1776	chr2:168788893-168788936	T	23	1		
	D13S317	chr13:82148025-82148088	NPTV	25	2	D13S317-54	rs73525369, G>A, 0.01
						D13S317-25	rs73250432, C>T. 0.02
	SE33	chr6:88277144-88277281	NV	30	6		
	D19S433	chr19:29926234-29926285	NPTV	31	1		
	TH01	chr11:2171086-2171113	NPTV	34	3		
	FGA	chr4:154587729-154587820	NPTV	56	2		
	D16S539	chr16:86352702-86352745	NPTV	60	1		
b	D22S1045	chr22:37140287-37140337	NPTV	16			
	PentaE	chr15:96831012-96831036	NPTV	16			
	D9S1122	chr9:77073818-77073873	NV	60			
	D17S1301	chr17:74684855-74684902	NV	97			
c	D18S51	chr18:63281668-63281756	NPTV	122			
	D1S1656	chr1:230769605-230769680	NPTV	122			
	D3S1358	chr3:45540737-45540800	NPTV	146			
	CSF1PO	chr5:150076325-150076376	NPTV	151			
	vWA	chr12:5983958-5984045	NPTV	163			
	D2S441	chr2:68011947-68011994	NPTV	175			
	D12S391	chr12:12297019-12297094	NPTV	178			
	D2S1338	chr2:218014859-218014950	NPTV	199 and within STR			
	D10S1248	chr10:129294244-129294295	NPTV	215			
	D6S1043	chr6:91740223-91740270	NTV	219			
	D4S2408	chr4:31302798-31302833	NTV	253			
	D5S818	chr5:123775553-123775596	NPTV	347			
	D6S474	chr6:112557951-112558018	T	385			

Autosomal STRs covered by commercial MPS kits. STR locations are based on STRNaming bracketing of ISFG Minimum Range in non-converted DNA. Minimum distance to CpG sites was calculated based on the forward strand. a) 18 STRs with at least one CpG site within 100 bp distance. b+c) Excluded 17 STRs due to inefficient STR amplification (b) or CpG sites more than 100 bp away from the STR locus (c). SNPs (global MAF >0.01) within CpG sites that might impact DNAm are listed. *MAF values from dbSNP build 156. *N* IDseek OmniSTR Global (NimaGen), *T* Precision ID GlobalFiler NGS STR Panel v2 (Thermo Fisher Scientific), *P* PowerSeq 46GY System (Promega), *V* ForenSeq MainstAY/Signature (Qiagen, formerly Verogen)

For the approach of simultaneous tDMP and iSNP analysis, four loci previously used in combined tDMP-iSNP analysis (cg20162146 and cg24742744 for semen, cg06379435 for blood, cg21189537 for saliva) were analyzed [13, 21, 23]. In addition, four tDMPs (cg24124443 for blood, cg21597595 for saliva, cg03874199-212 and cg26079753 for vaginal fluid) were chosen, as they harbor at least one iSNP (defined in this study as a SNP with a global major allele frequency (MAF) greater than 0.1) within 100 bp (Table 2) [9, 10, 28, 29].

**Table 2:** Selected regions for combined tDMP and iSNP analysis

CpG site (tDMP)				iSNP			
target ID	location (GRCh38)	target body fluid (DNAm levels)	distance tDMP to iSNP [bp]	target ID	location (GRCh38)	alleles	global MAF*
cg20162146	chr22:35399401	semen (low)	104	rs4645726	chr22:35399297	G>C	0.14
			19	rs1078979	chr22:35399420	A>G	0.35
cg24742744	chr2:126804511	semen (low)	27	rs12997574	chr2:126804484	A>C	0.37
cg06379435	chr19:3344276	blood (high)	6	rs72973031	chr19:3344282	C>T	0.13
cg24124443	chr12:107317713	blood (high)	12	rs68094061	chr12:107317725	A>T	0.17
cg03874199-212	chr2:176099515	vaginal fluid (high)	61	rs711812	chr2:176099576	C>A	0.34
cg26079753	chr12:53961745	vaginal fluid (high)	28	rs12826786	chr12:53961717	C>T	0.38
			78	rs874945	chr12:53961667	C>T	0.37
cg21189537	chr22:44159200	saliva (low)	52	rs79471	chr22:44159148	G>A	0.37
cg21597595	chr2:5366096	saliva (high)	45	rs17356301	chr2:5366141	T>C	0.12

*MAF values from dbSNP build 156

### Array-based characterization of CpG sites close to STR regions

As an initial approach, we checked whether STR-neighboring CpG sites were covered by the HumanMethylation450 and HumanMethylationEPIC BeadChip arrays (Illumina, San Diego, CA, USA), respectively. As STR-neighboring CpGs were only covered by 450 K BeadChip array, DNAm levels were evaluated using the processed, quality-filtered and normalized data from the NCBI Gene Expression Omnibus repository dataset GSE59509. This dataset includes 42 samples from blood, saliva, semen, vaginal fluid and menstrual blood [9].

### Panel design for simultaneous STR/iSNP and DNAm analysis using MPS

Primers for 22 STRs (cf. Table 1a+b) and eight tDMR-iSNP loci were designed with the Free Bisulfite Primer Design Tool (Zymo Research, CA, USA) and checked with BiSearch [30] and Genetools SNPCheck v3. Primers were designed to cover the full range of STR repeat regions based on STRNaming bracketing [2]. In cases of D3S4529, FGA, D8S1179, TH01, D14S1434, D16S539, D19S433 and PentaD the primers encompass the STR repeat region, but not the entire minimum reporting range that was recommended by the ISFG in 2024 [2]. SE33 primers cover the historical STR range but the forward primer extends 10 nucleotides into the ISFG minimum range and overlaps repeats that are displayed in brackets using STRNaming on the ISFG minimum range. To comply with STR repeats based on STRNaming brackets, two nucleotides of forward primer sequence are reported in the output for SE33. Two primer pairs for tDMP-iSNP loci were adopted or modified from previous publications (cf. Table S1). Prior to the analysis by MPS, the amplicons were verified by Sanger sequencing in singleplex reactions using the PCR primers for sequencing. PCR and Sanger sequencing were performed as described below. Specific and non-preferential amplification was verified using converted non-methylated and methylated control DNA in defined mixtures (Human Methylated and Non-methylated DNA Set, Zymo Research and EpiTect PCR Control DNA Set, Qiagen) with 0, 10, 25, 50, 75, 90 and 100% methylated DNA.

### Adaption of the FDSTools library file

A modified version of the library file within the FDSTools software package was necessary as a prerequisite for the MPS data analysis. The library file contains information about the analyzed targets, e.g. target location, reference sequence and STR repeat pattern. An adapted library file for bisulfite-converted DNA was developed to allow for allele calling in combination with DNAm determination for each read/read group.

To generate the library file, we prepared a pre-library file containing the STR and tDMP-iSNP target names, chromosome and primer loci (GRCh38) and specified the amplified DNA strand (forward or reverse), as the DNA strands are no longer complementary after bisulfite conversion (File S1). The construction of the final library file was the result of a Python script (File S2) executed on the pre-library file with Python v3.9, including the following steps:

1. Non-converted reference (forward or reverse orientation) and primer sequences were accessed based on chromosome and primer loci provided in the pre-library file.
2. The reference sequence was in-silico converted: Targeting the forward strand, all Cs in the reference were changed to T, if C was not followed by G. Targeting the reverse strand, all Gs in the reference were changed to A, if there was no C upstream of G. All CpG sites were expected to be methylated and remain as CG in the reference sequence. If CpG sites were present within the primers, CG were replaced with YG (targeting the forward strand) or CR (targeting the reverse strand). The final library file (.ini) (File S3) includes the sections [genome_position] and [flanks] for each STR and tDMP-iSNP target. The section [genome_position] contains chromosome and target loci up to the primer sequences that are given in the [flanks] section.
3. The STRNaming algorithm [3] was used to find repeat patterns in the reference sequences. Thus, for STR targets, the library file contains the [repeat], [prefix] and [suffix] sections, denoting the repeat structure and the 5’ and 3’ sequence flanking the repeat structure (up to the primer sequences), respectively. In addition, the [block_length] and [length_adjust] sections configure the STR repeat length and offsets to adjust STR allele numbers expected by CE. STR repeats and brackets are according to the ISFG recommendations [2]. For D2S1776 and PentaD, bracketing was manually adjusted to correspond to that of the ISFG-defined minimum range [2]. For D2S1776, AC[5]AGAT[1]AAAA[1] was moved from [repeat] to [prefix] to avoid flanking sequence alignment problems, as the AC repeat upon conversion to AT gets extended with an additional repeat. For PentaD, this was done by moving CGAAGGGG[1]A[7] from [repeat] into [suffix] so that the CpG site would not be in the middle of the bracketed repeat.
4. For tDMP-iSNP targets, no repeat patterns were recognized and their reference sequences (up to the primer sequences) are provided in the [no_repeat] section. For tDMR-iSNP targets, the chromosome locations of C (targeting forward stand) or G (targeting reverse strand) within each CpG site were listed in the [microhaplotype_positions], which lets FDSTools output the methylation state as a microhaplotype.

### STR/iSNP verification of control DNA

Four commercially available control DNA (2800M, 9947A (both Promega), 9948 (Qiagen), Human HCT116 DKO Non-Methylated DNA (from the Human Methylated and Non-methylated DNA Set, Zymo Research)) were used as controls for the assay and pipeline validation. STR alleles of non-converted DNA were provided by the manufacturers, extracted from STRBase and published research articles [31–34], as well as examined using the IDseek OmniSTR Global Autosomal STR Profiling Kit (Nimagen) according to the manufacturer’s protocol and data analysis published in [35]. The determination of alleles at selected iSNP positions was accomplished via Sanger sequencing of non-converted DNA.

### Sample collection

Biological samples were obtained from 48 volunteer donors (25 female, 23 male, age range 18-63 years, median age: 36.0 years). All participants gave informed consent, and the project is part of a larger study approved by the ethics committee of the University of Freiburg (22-1498-S1). Sample collection was performed by the participants themselves. All swab-based sampling included the use of DNA-free nylon FLOQ swabs (Copan, Murrieta, CA, USA). Blood (*n*=20) was taken from a finger prick, filled in a plastic capillary and applied on a swab. Semen (*n=*20) was collected in plastic cups, and a portion was directly absorbed on a swab. Vaginal fluid and nasal secretion (*n*=20 each) were obtained by careful rubbing using swabs. Menstrual blood (*n*=20) was collected on a swab within the first three days of the menstruation either from a menstrual cup, a sanitary napkin, or by dripping directly onto the swab. No sexual activity was allowed within 72 hours prior to collection of vaginal fluid and menstrual blood. Saliva (*n*=20) was spit onto QIAcard FTA Elute Indicating Micro Cards (Qiagen). Buccal mucosa (*n*=20) was obtained by rubbing at the inner surface of the cheek. Collected swabs were stored at −80 °C upon arrival until analysis, and FTA cards were stored at room temperature.

### DNA extraction, preparation of mixture and bisulfite conversion

DNA from blood, semen, vaginal fluid, menstrual blood, buccal mucosa and nasal secretion was extracted using the QIAamp DNA Mini Kit (Qiagen) according to the manufacturer’s protocol with minor adjustments: To enhance sperm lysis in semen swabs, 20 μL 1 M DTT (Promega) per sample was added to AL buffer. In case of blood and menstrual blood swabs, AW1 buffer was applied twice to enhance washing efficiency. DNA from saliva cards was extracted using the QIAamp DNA Micro Kit (Qiagen) following the manufacturer’s protocol. Half of the saliva cards were cut into pieces and put into a Lyse&Spin basket (Investigator Lyse&Spin Basket Kit, Qiagen). DNA was eluted in 50 μL DNA-free water. Extracted DNA was quantified using the Power Quant System (Promega) on a QuantStudio 5 System with QuantStudio Design and Analysis Software v1.5.2 (TFS).

For the preparation of a two-person mixture, 25 ng of extracted semen and saliva DNA each was mixed in a 1:1 ratio prior to bisulfite conversion.

Bisulfite conversion was conducted using 50 ng DNA or a maximum of 30 μL with the EZ DNA Methylation Gold Kit (Zymo Research) according to the manufacturer’s protocol. DNA was eluted in 15 μL M-Elution buffer. Single-strand DNA (ssDNA) quantity and conversion efficiency were determined using a modified version (without FLJ39739) of the multiplex quantitative real-time PCR system BisQuE [16]. Reactions were run on the QuantStudio 5 System. Data was analyzed using the BisQuE data analysis sheet as provided in [16].

### Singleplex PCR and Sanger sequencing

Successful amplification and specificity of primers for bisulfite MPS and iSNP alleles in non-converted DNA were verified using Sanger sequencing. Singleplex PCR was performed using 2.5 μL PyroMark Master Mix (Qiagen), 0.6 μL Coral Load (Qiagen), 0.5 μg BSA (TFS), 1 μL of the 2.5 μM forward/reverse primer mix (Table S1), 1 μL of (bisulfite-converted) DNA (approx. 1 ng), and DNA-free water ad 6.5 μL. PCR cycling was conducted in a GeneExplorer 48/48 Gradient cycler (BIOER/Biozym, Germany) under the following conditions: 15 min at 95 °C; 40 cycles: 45 sec at 94 °C, 30 sec at 54 °C, 30 sec at 72 °C; final elongation for 10 min at 72 °C. PCR products were enzymatically purified using 1 μL ExoSAP-IT (TFS) per 5 μL PCR product under the following conditions: 45 min at 37 °C, 15 min at 80 °C. Sequencing was conducted using 1 μL BigDye™ Terminator 3.1 Ready Reaction Mix, 0.5 μL BigDye Sequencing Buffer (Applied Biosystems/TFS), 1 pmol forward or reverse PCR primer, 1 μL PCR product, and DNA-free water ad 5 μL. Sequencing cycling was conducted under the following conditions: 1 min at 96 °C; 25 cycles: 10 sec at 96 °C; 5 sec 50 °C; 4 min at 60 °C. The 96-DyeEx kit (Qiagen) was used for purification according to the manufacturer’s standard protocol before the sequencing products were run on the 3130xl Genetic Analyzer using POP-6 with data collection software (both Applied Biosystems/TFS) and analysis using the Sequencher DNA Sequence Analysis v5.4.6 Software (Gene Codes Corporation, Ann Arbor, MI, USA).

### Multiplex PCR and massively parallel sequencing (MPS)

The final 18 STRs and eight tDMP loci were subdivided in five multiplex PCR pools (A-E) and one singleplex PCR reaction (pool F: D5S2800) (Table S1). Multiplex PCRs (incl. D5S2800) were performed each using 7.5 μL PyroMark Master Mix (Qiagen), 1.5 μL Coral Load (Qiagen), 1.16 μg BSA (TFS), 2 μL of the multiplex primer mix, 1-2.5 μL of bisulfite-converted DNA (approx. 1 ng to reflect common DNA amounts used for PCR), and DNA-free water ad 15 μL. PCR cycling was conducted under the following conditions: 15 min at 95 °C; 15 cycles: 45 sec at 94 °C, 30 sec at 54 °C, 30 sec at 72 °C; 25 cycles: 45 sec at 94 °C, 30 sec at 62 °C, 30 sec at 72 °C; final elongation for 10 min at 72 °C. The six PCRs per sample were processed separately up to equimolar pooling (see below). PCR products were cleaned using 1.9-fold volume magnetic beads (GE Healthcare, Little Chalfont, UK; pre-treated and purified according to [36]). The second PCR for attachment of adapters/indices was carried out in a 12 μL-volume using 1 μL PCR product, 6 μL NEBNext Ultra II Q5 Master Mix (New England Biolabs, Ipswich, MA, USA), 5 pmol of Nextera XT i5- and i7 index primer each and 3 μL of DNA-free water. PCR cycling was conducted under the following conditions: 30 sec at 98 °C; 6 cycles: 10 sec at 98 °C, 30 sec at 62 °C, 45 sec at 65 °C; final elongation for 5 min at 65 °C. PCR products were cleaned using 1.6-fold volume of magnetic beads and eluted in 15 μL. Products were quantified using the dsDNA high-sensitivity Qubit Quantification Kit (TFS) and were equimolar pooled. The final 10.5 pM library including 20 pmol phiX was sequenced using 350/150 bp cycles on a MiSeq FGx (Verogen) using Miseq Reagent 500 bp (micro) kits (Illumina).

### MPS data analysis

**Fig. 1:**
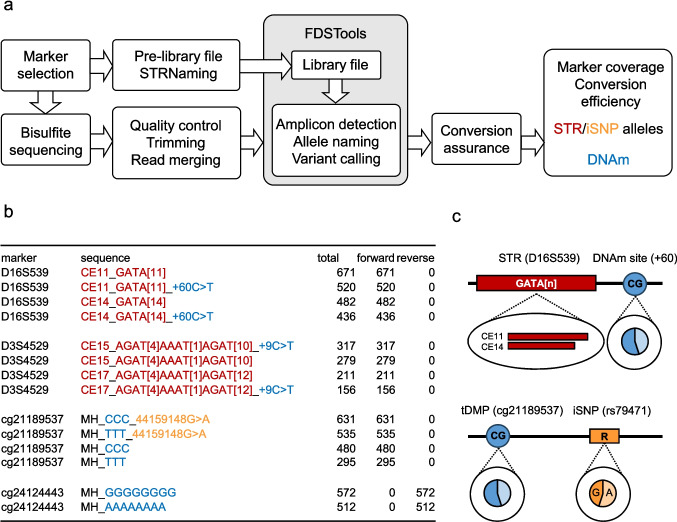
Combined analysis of bisulfite-converted STR and iSNP targets using MPS and FDSTools. **a** Workflow of bisulfite sequencing data analysis. Prior to sequencing, the final library file for bisulfite-converted DNA required for FDSTools package was generated based on the pre-library file. Raw bisulfite sequences are processed (quality check, trimming, merging) before FDSTools packages combined with STRNaming algorithm are used for amplicon detection, allele naming and variant calling (including non-methylated DNAm sites and iSNPs). Conversion assurance is conducted prior to evaluation of marker coverage, conversion efficiency, STR/iSNP allele and DNAm. **b** Output of FDSTools seqconvert tool (1:1 mixture non-methylated and methylated control DNA). For STR targets, CE-based STR alleles, repeat structure based on ISFG recommendations (red) and flanking variants (blue), including non-methylated DNAm sites, are listed. TDMP-iSNP targets are treated as microhaplotypes and the output contains lined up bases at each CpG within amplicon (blue), location and transition of flanking variants, including alternative alleles at iSNP sites (orange). Column “total” provides the number of reads for each unique sequence and read counts in forward or reverse columns report the amplified bisulfite converted DNA strand. **c** Based on FDSTools output, read counts of STR (red) and iSNP (orange) genotypes, methylated and non-methylated reads at DNAm sites (blue) can be extracted. R: Guanine or adenine

The obtained fastq files were first processed using the following packages and steps on a locally installed version of the Galaxy server platform [37]. Reads were trimmed using Trim Galore! v0.6.7 [38] for adapter trimming and quality control under the following parameters: Phred quality score threshold for trimming low quality ends = 20, maximum average expected errors = 0.01, minimum read length = 50. Paired-end reads were merged with FLASH v1.2.11.4 [39] using the following settings: Minimum read overlap = 30 bp, maximum read overlap 150 bp, maximum mismatch density = 0.33 (Fig. 1a).

For amplicon detection, allele naming and variant calling, tools from the software package FDSTools v2.1.1 [40] combined with algorithm STRNaming v1.2.0 [2, 3, 41] and the adapted library file were executed in Powershell with Python. First, tssv tool with arguments minimum = 1 (no cutoff, all unique reads are reported) and mismatches = 0.05 (number of mismatches (per nucleotide of flanking sequences) allowed in flanking sequences to identify targets) was used to link raw reads to the targets (analyzed forward or reverse strand) and count the numbers of reads for each unique sequence. The FDSTools seqconvert tool was used afterwards to convert raw sequences into allele names (format = allelename) and to detect flanking variants, including C>T and G>A transitions caused by bisulfite conversion of non-methylated Cs.

Conversion assurance, marker coverage, conversion efficiency checking, STR and iSNP allele evaluation, DNAm analysis and data visualization was conducted in JupyterLab v3.6.3 (Anaconda Navigator) using pandas v2.0.3, pingouin v0.5.4 and seaborn v0.12.2 packages (Fig. 1a). For conversion assurance, non-converted reads (with no conversion at CpH sites, i.e., C not followed by G) were excluded from the analysis. Reads from tDMP-iSNP loci with “N” at CpG sites were removed, as they indicate an uncertain read mapping due to an immediately flanking InDel. This only occurred in a small number of reads. For reliable DNAm analysis, the threshold for the number of mapped read pairs was set to 800 (corresponding to the analysis of 800 molecules). A mean conversion efficiency of 99.7% (98.2-100%) was obtained for all markers across all samples. In order to evaluate allele-linked DNAm levels, reads were clustered based on STR/iSNP alleles subsequent to conversion efficiency checking.

## Results and discussion

### STR selection and array-based characterization of candidate regions neighboring STRs

Initially, 35 autosomal STR loci were selected based on their presence in commercially available sequencing kits (Table 1). Twelve STRs were removed as they did not contain any CpG sites within either 100 bp flanking region. The flanking region was restricted to 100 bp to allow for successful analysis of degraded DNA. D2S1338 contains a CpG site within the reference repeat pattern (GGAA[2]GGAC[1]GGAA[n]GGCA[n]). However, at least three alternative sequence patterns without the CpG site exist (GGAA[n]GGCA[n], GGAA[n]GAAA[1]GGAA[2]GGCA[7] and GGAA[n]GGGA[1]GGCA[7]), therefore D2S1338 was consequently removed from the panel (Table 1c).

For a first impression of tissue-specific differences, we analyzed STR-neighboring sites using publicly available data of DNAm arrays. Among the 22 STRs, only two neighboring CpG sites are part of the Illumina 450 K and none on the EPIC BeadChiP Array, limiting analysis to two CpG sites. Beta values from the dataset GSE59509, containing forensic relevant body fluids, were analyzed for differences (Table S2, Fig. S1). TPOX flanking cg00626390 shows high DNAm in all tested body fluids with low inter-individual variation (highest variation in saliva with standard deviation (SD)=4.3%). The CpG site near SE33 (cg10502590) shows DNA hypomethylation levels (mean=31.2%, SD=8.3%) in semen samples. Saliva, blood, vaginal fluid and menstrual blood are highly methylated in the SE33 flanking CpG, with highest variation in saliva samples (SD=8.0%). A known C>T transition at the cg10502590 position (rs189881506, global MAF=0.0029) might affect the cytosine methylation [42]. In case of a heterozygous genotype (C/T), the DNAm can then only reach a maximum of 50% and no DNAm occurs in case of a homozygous T/T genotype. The presence of the SNP cannot be discriminated from a bisulfite-converted non-methylated cytosine if the forward strand is analyzed, as that also leads to a C>T change. However, the analysis of the reverse strand (G>A) allowed identification of the SNP. An initial characterization with publicly available array data shows that SE33 flanking cg10502590 might be a promising marker for semen contributor assignment.

### Bisulfite sequencing of STRs and selected tDMP and iSNP sites

Primers were designed for 22 STRs (Table 1a+b). In case of STRs with CT-rich sequence patters (D1S1677, SE33, D7S820, D8S1179, D14S1434, D19S433, D21S11) the reverse strand was analyzed to avoid sequencing of long T stretches. The PCR amplicon of D19S433 does not contain any cytosine with a downstream non-guanine base (CpH) to verify bisulfite conversion. Successful DNA conversion was verified via BisQuE [16] and by checking all primers beforehand for specific binding to only converted DNA. Efficient amplification of D9S1122, D17S1301, D22S1045 and PentaE in bisulfite-converted DNA was not possible in this study, even after testing different primers and primer combinations (Table S1), thus resulting in the removal of these four markers (Table 1b). In total, 18 STRs with 38 neighboring CpG sites (one to six per amplicon) were selected for MPS analysis (Table 1a). For six flanking CpG sites, possible changes due to a SNP (global MAF >0.01) could affect DNAm. Therefore DNAm analysis of CpG sites D5S2800+21 (rs12187142), D7S820+10 (rs16887642), D13S317-54 (rs73525369), D13S317-25 (rs73250432), D14S1434-2 (rs112858516) and D20S482-12 (rs77560248) require special attention as measured DNA hypomethylation levels can be caused by a non-methylated cytosine or a SNP [29].

To design PCR amplicons for simultaneous tDMP and iSNP analysis as short as possible, markers were selected based on body fluid-distinct DNAm and iSNPs with global MAF >0.1 in close proximity [29]. When analyzing SNPs in bisulfite-converted DNA, the base transition and the amplified DNA strand (forward or reverse) have to be considered. As mentioned before, nucleotides with a C>T or T>C transition cannot be distinguished after bisulfite treatment when analyzing the forward strand (as they appear in both cases as a T, unless a new CpG site with potential DNAm is created). In the case of the iSNPs near cg06379435 (rs72973031), cg21597595 (rs17356301) and cg26079753 (rs12826786 and rs874945), we amplified the reverse strand and detected complementary alleles A and G. The iSNP rs72973031 (G>A) is part of a CpG site, therefore DNAm at the location of rs72973031 (cg06379435+6) was not included in the analysis. The alternative allele of rs4645726 (G>C) generates a CpG site. The C can be methylated or non-methylated, consequently the alternative iSNP allele can be detected as C or T. The alleles of rs711812 (C>A) are detected after bisulfite treatment as T or A. Summarizing, ten iSNPs were analyzed in the region of eight known tDMP markers (Table 2).

### Combined STR/iSNP and DNAm analysis

Tools of the FDSTools package and STRNaming algorithm were used with a modified library file for STR allele reporting of bisulfite converted DNA as well as linked STR, iSNP and DNAm analysis (Fig. 1a). First, merged read pairs were assigned to specified targets and the number of reads for each unique sequence was counted. Second, allele names, STR flanking variants, DNAm status at CpG sites, and alternative iSNP alleles are compiled in a compact output (Fig. 1b).

The modified library file which forms the basis of FDSTools software packages for identifying targeted amplicons, allele calling and DNAm determination analysis is however not restricted to the aim presented in this study. Adaption might be possible for other STR-calling tools used in the forensic community by implementation of modified libraries/panels for STR and flanking region analysis of bisulfite converted DNA. In contrast, other freely available tools e.g. MethHaplo, SNPsplit, MethlyGenotyper and Bis-SNP are available for read clustering based on SNPs, microhaplotypes or DNAm but none of these tools currently converts highly variable STR sequences in reporting format according to the ISFG guidelines or link DNAm to complex STR alleles in bisulfite-converted DNA [43–46].

For STR targets the FDSTools output file contains the expected CE-based allele (CEx_) and the allele repeat pattern (comprised of STR in forward orientation following ISFG recommendations [2]) (both marked red in “sequence” column in Fig. 1b). All cytosines in STR-flanking CpG sites are expected to be methylated in the reference. Otherwise they appear after bisulfite conversion as C>T or G>A (reverse strand) reference deviation along with distance to STR (blue in Fig. 1b) according to the STRNaming-defined bracketed repeat. We performed bisulfite sequencing of a 1:1 mixture of non-methylated and methylated control DNA (Human Methylated and Non-methylated DNA Set, Zymo Research) and display FDSTools output of exemplary targets in Fig. 1b. D16S539 consists of alleles 11 and 14 with the repeat pattern GATA[11] and GATA[14]. A non-methylated CpG site 60 bp downstream of D16S539 (+60C>T) was found in 520 merged read pairs on the same read as allele 11. 671 reads were according to the reference and represent methylated cytosines, thus resulting in an allele 11-linked DNAm of 56%. Reads are only found in the orientation (forward or reverse) of the initially amplified bisulfite-converted DNA strand. Total DNAm levels of D16S539+60 were calculated as follows: (Total D16S539 reads – D16S539 reads containing “+60C>T”)/total D16S539 reads (Fig. 1c).

For the included tDMP-iSNP targets, the FDSTools library file was modified (cf. Material and Methods) to contain the chromosome locations of all CpG sites within the included amplicons, instructing FDSTools to treat and display these CpG sites as microhaplotypes (Fig. 1). In the “sequence” column the bases of specified CpG locations are lined in a row and represent the amplicons methylation haplotype (blue). C (analyzing bisulfite-converted forward strand) and G (analyzing bisulfite-converted reverse strand) represent methylated cytosines, correspondingly T and A represent non-methylated cytosines. If the sequenced bases differ from the reference e.g., the iSNP consists of an alternative allele, these variations are listed together with the chromosome position in forward orientation (orange in Fig. 1c). To address allele-linked DNAm levels, reads were clustered based on iSNPs (orange) prior to DNAm estimation. As an example, in cg21189537, 631 (or 535) merged reads report all CpG sites (cg21189537 plus two neighbor sites) within the amplicon to be methylated (or non-methylated) in combination with the presence of the alternative allele A in iSNP rs79471 (chr22:44159148). In consequence this leads to 54% DNAm next to allele A in iSNP rs79471 and 62% for allele G in the 1:1 control DNA mixture. Overall DNAm levels per sample were calculated per CpG site, by determining the ratio of methylated reads (C or G) against the total number of reads (C+T or G+A) (Fig. 1c).

Attention must be paid when evaluating STR loci D12ATA63 and D2S1776. D12ATA63 consists of repeat pattern TGT[4]TAT[n] in STRNaming-defined bracketing followed by TAC in the flanking region. After bisulfite conversion the STR downstream bases TAC generate an additional TAT repeat in the analyzed sequence. If the +18C>T variant (+18 CpG non-methylation) occurs, difficulties arise in suffix and STR boundary detection, resulting in STR structure compensation by +2ATT>- (Fig. S2). In D2S1776, we observed minus 2 bp, minus 1 repeat (4 bp) and plus 2 bp stutter artefacts after MPS, presumably resulting from the AC[5] repeat (converted to AT[5] repeat) 14 bp upstream of the STRNaming bracketing (Fig. S3). Attention must also be paid to T>C (bisulfite-converted forward strand) and A>G (bisulfite-converted reverse strand) variants in CpH sites as an indicator for incomplete bisulfite conversion of the sample.

### Verification of STR and iSNP alleles

Four commercially available control samples (2800M, 9947A, 9948, HCT116 DKO) were analyzed in duplicates to verify correct allele calling of STRs and iSNPs in bisulfite converted DNA (Table S3a-d). The most common STR alleles and their read counts were extracted and compared to available length-based reference data of the control DNA. Reference data for verification of the results was not available for all STRs (non-colored).

In 45 out of 46 heterozygous genotypes in the four control DNA, the two alleles were correctly called with the highest read number matched to the length-based reference alleles (green in Table S3a-d) in both replicates. Large discrepancies in length between heterozygous alleles in SE33 presumably contribute to imbalanced profiles as observed in 9947A (replicate 2: CE19: 3749, CE29.2: 620) and HTC116 DKO (replicate 1: CE14: 5105, CE26.2: 881; replicate 2: CE14: 6325, CE26.2: 970). In replicate 1 of 9947A, we observed that the stutter (CE18: 675) of the shorter allele (CE19) even exhibited a greater number of reads than the longer allele (CE29.2: 477) (orange in Table S3a). This suggests that the shorter allele may be preferentially amplified, and/or the longer allele partially degraded during bisulfite conversion [47, 48]. Furthermore data analysis might be hampered due to length and sequence structure of SE33.

In all homozygous STRs, the reference allele was correctly identified as the allele exhibiting the greatest number of reads. A closer look at the second most common allele identified all of them as minus 1 repeat stutter, minus 1 bp or minus 2 bp stutters (marked with an asterisk in Table S3). The highest stutters were measured with 21.1% in D21S11 of 9947A (all stutters >20% blue in Table S3) are thus within the normal range of STR analysis using MPS [49–51] (21%). It must be noted that D21S11 includes multiple stutter variants. For example, the allele 30 of 9947A contains two long uninterrupted repeats (14 and 12 copies respectively, allele TCTA[14]TATC[4]A[1]TCTA[2]TCCA[1]TATC[12], cf. Fig. S4). The stutter artefact CE29 is composed of stutters of the first (TCTA[13]TATC[4]A[1]TCTA[2]TCCA[1]TATC[12]) and the second repeat (TCTA[14]TATC[4]A[1]TCTA[2]TCCA[1]TATC[11]) (Fig. S4). With an enlarged sample size, stutter filtering and correction features of FDSTools (stuttermark, stuttermodel) can be used to accurately measure the expected stutter proportions. The methylation status is also reflected in the stutter artefacts.

Previous MPS approaches have identified an isoallele 13 in D8S1179 of control DNA 9947A [31, 32, 34]. Due to the CT-rich STR pattern (TATC[n] TGTC[n] TATC[n]) of D8S1179, we analyzed the reverse strand in bisulfite sequencing (non-converted GATA[n] GACA[n] GATA[n]). After bisulfite sequencing the isoallele information of GATA[10] GACA[1] GATA[2] is lost and both alleles were sequenced as GATA[13] and reported on the forward strand as TATC[13].

For iSNPs, we calculated the numbers of reference, minor allele reads, and allele read ratio, and verified the results by Sanger sequencing of non-converted DNA (Table S4). As the homozygous genotypes show only few reads of the other allele (allele read ratio maximum 0.02) and the heterozygous allele reads show a balanced profile (allele read ratio between 0.29 and 0.5), the iSNP genotypes were correctly identified in all samples (green in Table S4).

Our results reveal promising analysis of STRs and iSNPs in bisulfite-converted DNA. Furthermore, the STR analysis of bisulfite-converted DNA provides the opportunity to include STR amplicons generally in DNAm tests for other applications, thereby preventing mix-ups during sample processing. Such sample tracking tools are important for diagnostic tests that use additional analysis of iSNP markers [52]. Difficulties were identified in distant heterozygous alleles when the shorter allele is preferentially amplified (as observed for SE33). Caution must be paid on the imbalance of heterozygous alleles, stutters and potential loss of isoallele information (e.g., in D8S1179).

Future work will concentrate on integrating STR sequence variation and flanking SNPs into the analysis to increase allele information. In addition, FDSTools stutter models will be trained similar to those previously described for non-converted STRs [40].

### DNAm of STR-neighboring CpG sites

**Fig. 2:**
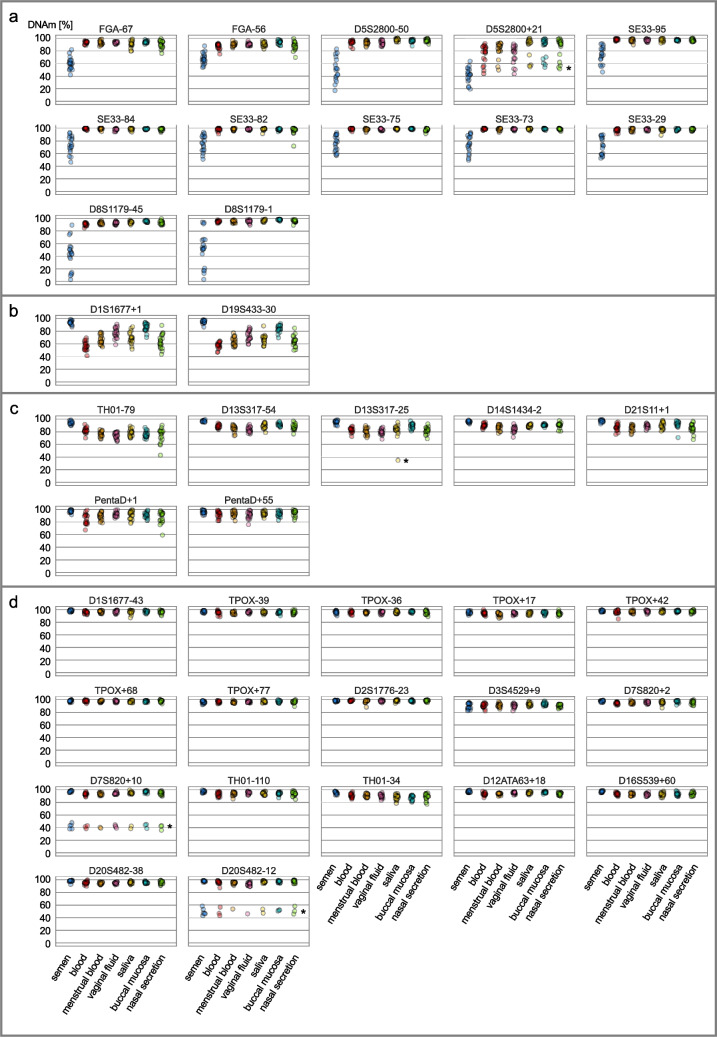
Individual DNAm values of STR neighboring CpG sites in different body fluids. DNAm values of all investigated STR neighboring CpG sites (- indicates upstream of STR, + downstream) obtained from bisulfite MPS analysis in semen (blue), blood (red), menstrual blood (orange), vaginal fluid (pink), saliva (yellow), buccal mucosa (turquoise) and nasal secretion (green). SNP-caused DNA hypomethylation are marked (*). CpG sites were grouped based on differential DNAm levels in semen samples (**a**), tissue-differential DNAm (**b**), small inter- and intra-body fluid varying DNAm (**c**) and absence of apparent DNAm differences between individuals and body fluids (**d**)

We characterized the DNAm of 38 CpG sites neighboring 18 STRs. Twenty samples of each body fluid (semen, buccal mucosa, vaginal fluid, blood, saliva, menstrual blood and nasal secretion), from a total of 48 different individuals, were analyzed for that purpose. Individual DNAm values as well as mean DNAm values and standard deviation per body fluid were evaluated for each marker (Fig. 2, Table S5).

We identified tissue-independent DNA hypomethylation levels (35.1-74.6%) in several individuals in D5S2800+21 (*n*=9), D7S820+10 (*n*=9), D13S317-25 (*n*=1) and D20S482-12 (*n*=7) (marked with an asterisk in Fig. 2). All of these individuals harbor SNPs, which could not be discerned from non-methylated sites after bisulfite conversion (Table 1a). Sanger sequencing of non-converted DNA revealed that heterozygous SNPs limited the possibility of methylation in these samples due to loss of the CpG site (data not shown). In the future vision of case work implementation, it is recommended that for each STR/iSNP locus sequenced in converted DNA, the same loci should be sequenced in non-converted DNA to identified potential SNPs in neighboring CpG sites. The DNAm values of these individuals were excluded prior to the calculation of the mean DNAm and the standard deviation (Table S5).

In our analysis, FGA (−67, −56), D5S2800 (−50, +21), SE33 (−95, −84, −82, −75, −73, −29) and D8S1179 (−45, −1) flanking CpG sites have shown differential DNAm levels in semen compared to the other body fluids (Fig. 2, group a). Some semen donors display DNA hypomethylation down to 2.9% (D8S1179-45), while others display DNA hypermethylation that overlaps with non-semen samples. In D8S1179-45 and D8S1179-1 we observed allele associated DNAm levels, forming the three-part pattern around 10%, 50% and 90%. For instance, semen donor 7 shows a total 47.1% DNAm in D8S1179-45 with 5.2% and 89.5% DNAm assigned to alleles 11 and 15, respectively (Table S6). Previous study of genome-wide STRs and DNAm found correlations between STR allele and DNAm levels at specific loci [53]. However, more samples with different repeat structures are needed to confirm this hypothesis. Higher alleles (allele 14 and 15) of D8S1179 do not exhibit lower DNAm levels specific to semen in D8S1179-45 and D8S1179-1. Additional samples are needed to determine allele-specific cutoff values for semen identification. No other explanation for the observed pattern due the donor’s chronological age, SNPs at the DNAm sites, and technical causes (coverage, bisulfite conversion efficiency) was found.

Both D1S1677+1 and D19S433-30 display different DNAm levels between the body fluids, with highest mean DNAm in semen (93.9% and 95.0%) and lowest mean DNAm in blood samples (56.9% and 57.8%) (Fig. 2, group b). The analysis of individual DNAm values shows interindividual heterogeneity. One reason might be the varying leukocyte (or sub-cell type) content of these fluids.

TH01-79, D13S317-54, D13S317-25, D14S1434-2, D21S11+1, PentaD+1 and PentaD+55 show mean DNAm levels >75.1% in all body fluids with slight differences (Fig. 2, group c). However, they display overlap between body fluids and broad variance within body fluids, e.g. variance in nasal secretion samples in TH01-79 (mean=75.1%, SD=11.1%). Consistently high DNAm levels (mean >87.7%) and barely any variance within body fluids was measured near D1S1677 (−43), TPOX (−39, −36, +17, +42, +68, +77), D2S1776 (−23), D3S4529 (+9), D7S820 (+2, +10), TH01 (−110, −34), D12ATA63 (+18), D16S539 (+60) and D20S482 (−38, −12) (Fig. 2, group d). For the investigated body fluids, we conclude that STR neighboring CpG sites in group c and d are not only insufficient for body fluid determination but also not informative for contributor assignment.

Examined DNAm levels of TPOX-36 (cg00626390) and SE33-82 (cg10502590) are in concordance with results obtained from 450 K BeadChip Array (Table S2). CpG sites neighboring FGA, D5S2800, SE33, D8S1179, D1S1677, and D19S433 (Fig. 2, group a+b) reveal different DNAm levels between body fluids. However, these sites alone are not sufficient for body fluid determination, but have to be further characterized to determine their potential in the assignment of body fluid to contributors in specific case scenarios (cf. mixture example below) and other applications.

DNAm analysis in and around STRs received minimal attention in the past. However, advancements in technology and the optimization of sequencing methods for repeat sequences have led to a growing interest of STR regions beside their use in forensic identification [54]. More samples are required to investigate whether STR allele (D8S1179), cell type composition within a given body fluid, chronological age, disease or lifestyle could cause inter-individual differences in DNAm levels in STR systems [10, 12, 55]. Consequently, the amount of accessible data and knowledge will increase in the future, resulting in a more comprehensive perspective on DNAm within tandem repeat regions.

### Evaluation of known tDMPs with flanking iSNPs

We evaluated the DNAm of eight published tDMPs with iSNPs in close proximity [13, 21, 23, 28]. Previous publications had shown DNAm only in the intended type of body fluid with a low standard deviation between individuals [9, 10, 28, 29]. The DNAm levels of some of these markers has not yet been investigated in menstrual blood, nasal secretions, and buccal mucosa (Table 3).

**Table 3:** DNAm evaluation of eight tDMPs for combined tDMP and iSNP analysis

	Mean DNAm values [%] ± SD						
CpG site (tDMP)	semen	blood	menstrual blood	vaginal fluid	saliva	buccal mucosa	nasal secretion
cg20162146	**8.0 ± 10.6**	95.2 ± 3.7	97.5 ± 1.9	96.4 ± 2.5	96.6 ± 2.8	95.6 ± 2.8	97.4 ± 2.8
cg24742744	**3.4 ± 4.1**	95.5 ± 3.3	95.1 ± 2.8	94.7 ± 3.0	95.5 ± 2.3	95.1 ± 2.7	96.0 ± 2.6
cg06379435	1.4 ± 2.0	**36.2 ± 6.9**	13.4 ± 7.8	4.4 ± 7.6	2.4 ± 1.9	1.0 ± 1.0	4.9 ± 4.9
cg24124443	0.6 ± 1.0	**18.6 ± 6.2**	6.4 ± 5.5	3.5 ± 2.4	4.1 ± 3.2	5.8 ± 4.3	3.5 ± 3.0
cg03874199-212	1.0 ± 1.3	4.7 ± 3.6	15.8 ± 11.0	**42.8 ± 18.2**	5.4 ± 3.8	10.2 ± 4.5	4.0 ± 2.9
cg26079753	1.0 ± 1.1	2.5 ± 1.5	22.9 ± 11.7	**48.4 ± 16.8**	2.4 ± 1.9	2.6 ± 1.4	1.3 ± 0.8
cg21189537	97.4 ± 1.5	97.8 ± 1.1	92.4 ± 4.3	94.9 ± 3.0	**64.9 ± 15.4**	21.2 ± 14.6	94.9 ± 2.9
cg21597595	0.6 ± 0.6	1.0 ± 1.1	2.9 ± 2.4	4.9 ± 4.1	**37.2 ± 12.4**	68.9 ± 7.3	9.4 ± 10.1

Mean DNAm values of tDMPs for semen, blood, menstrual blood, vaginal fluid, saliva, buccal mucosa and nasal secretion samples (n=20 each). Differential DNAm in target body fluids are indicated in bold

Both semen-specific DNAm markers (cg20162146 and cg24742744) show low DNAm levels (8.0% and 3.4% on average) in semen compared to other body fluids tested. It should be noted that no seminal fluid of vasectomized men was tested in this pilot study. Previous studies have shown that the DNAm profile of sperm-free seminal fluid can differ from semen of non-vasectomized donors, likely caused by differences in cell type composition [9]. DNAm markers for blood (cg06379435, cg24124443), vaginal fluid (cg03874199-212, cg26079753) and saliva (cg21189537, cg21597595) show intermediate DNAm levels in the target body fluids (mean DNAm 18.6% - 64.9%). Non-targeted body fluids show higher (cg21189537) or lower (others) DNAm levels in these markers. Menstrual blood samples show intermediate DNAm levels in one blood-specific (cg06379435) and both of the vaginal fluid-specific (cg03874199-212, cg26079753) markers. In these markers menstrual blood shows DNAm levels between targeted and non-targeted body fluids. Such results have been previously observed in other blood- and vaginal fluid-specific DNAm and mRNA markers [9, 10, 56]. In the blood-specific marker cg24124443 the level of DNAm in menstrual blood was similar to DNAm levels in vaginal fluid, saliva, buccal mucosa and nasal secretion. In the saliva-specific markers, buccal mucosa samples exhibited DNA hypomethylation (cg21189537) and DNA hypermethylation (cg21597595) and were more divergent from the non-targeted body fluids (semen, blood, vaginal fluid, menstrual blood, and nasal secretion) compared to saliva samples. As a previous study has shown, the method of saliva collection can greatly influence DNAm levels [57]. Nasal secretion samples show no cross-reaction with any of the markers tested, as previously published for cg21597595 and cg26079753 [58].

To date, simultaneous analysis of mRNA, miRNA and neighboring coding SNPs (cSNPs) has been studied more intensively than tDMP-iSNP sites. Several research groups have analyzed RNAs for blood, semen, saliva, vaginal fluid, menstrual blood, and skin cell identification and nearby cSNPs via MPS or CE-based assays and successfully used these targets to assign contributors to body fluids in mixtures [59]. The current research focuses on the identification of new mRNA/miRNA/circRNA and cSNP loci, sensitivity and mixture ratio testing. However, DNA-RNA co-extraction is not routinely used in most laboratories, presence of ubiquitous RNases poses a risk of rapid degradation, and cSNPs may affect the stability or expression of RNA transcripts, so genotypes may not match at the DNA and RNA levels [6].

### Allele-linked DNAm in an exemplary body fluid mixture

**Fig. 3:**
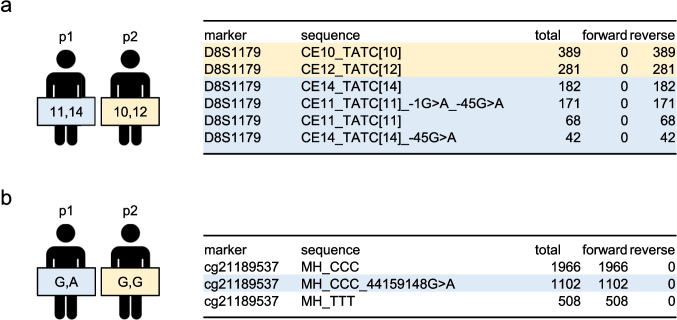
FDSTools output of STR and tDMP-iSNP targets in two-person-mixture. MPS analysis of two-man semen and saliva mixture (1:1 mixtures based on DNA amount in extract). Known reference alleles of mixture contributors and exemplary FDSTools output of D8S1179 (**a**) and cg21189537 (**b**) are depicted. Based on known reference alleles, reads can be linked to mixture contributor p1 (blue) or p2 (yellow). In the case of mixture contributors sharing alleles (G in rs79471) reads were non-colored

To perform a pilot test exploring the general feasibility of the assignment of contributor to specific body fluids, the analysis of a two-man-mixture of semen and saliva DNA extracts (1:1 mixture ratio) was conducted as the question might be, who of the two men contributed semen. In a crime case scenario, the mixture contributors, p1 and p2, would have already been identified using STR (and iSNP) profiling, and the presence of semen and saliva determined by using presumptive tests, RNA or DNAm analysis. In subsequent data interpretation, we integrate knowledge of the identified body fluids, information about two mixture contributors and their STR/iSNP reference alleles, as well as the mixture ratio. Total DNAm and STR/iSNP allele-linked DNAm was determined in D8S1179-45, FGA-67, SE33-73, D1S1677+1, D19S433-30, cg21189537, cg20162146, and cg21597595 as these CpG sites have shown distinct DNAm levels between semen and saliva and the contributors harbor distinct STR/iSNP genotypes (Fig. 3, Table 4). We interpreted allele-linked DNAm levels as (non-)semen/saliva indicative, if STR/iSNPs alleles could be exclusively assigned to one of the mixture contributors and display semen- or saliva-like DNAm levels (Fig. 2). Allele-linked (exclusively p1 or p2) DNAm levels non-indicative for semen and saliva were labeled as ‘inconclusive’. DNAm of shared alleles were not interpreted (marked with an asterisk in Table 4).

**Table 4:** Allele-linked DNAm analysis in body fluid mixture

	total DNAm [%]	allele-linked DNAm [%]			
		p1		p2	
D8S1179		11	14	10	12
D8S1179-45	80.3	36.3 semen	83.9 inconclusive	92.3 non-semen	92.6 non-semen
FGA		22	23*	19	23*
FGA-67	79.6	71.4 semen	74.0	95.0 non-semen	74.0
SE33		15.2	19	25.2	30.2
SE33-73	94.0	90.8 inconclusive	91.3 inconclusive	98.6 inconclusive	97.4 inconclusive
D1S1677		13*	15	13*	18
D1S1677+1	79.5	78.8	87.1 inconclusive	78.8	76.4 non-semen
D19S433		13	14*	14*	15
D19S433-30	78.7	92.6 inconclusive	74.5	74.5	67.4 non-semen
rs79471		G*	A	G*	G*
cg21189537	85.7	80.0	99.6 non-saliva	80.0	80.0
rs1078979		A*	A*	A*	G
cg20162146	71.8	51.8	51.8	51.8	98.8 non-semen
rs17356301		T	T	C	C
cg21597595	24.6	0.6 non-saliva	0.6 non-saliva	41.6 saliva	41.6 saliva
assigned body fluid		semen		saliva	

Total DNAm and allele-linked DNAm levels were estimated based on the FDSTools output of a two-man (p1 and p2) mixture of semen and saliva (Fig. 3). Reads were clustered based on known STR/iSNP genotypes (alleles underlined), before allele-linked DNAm was estimated. DNAm levels are labeled as (non-)semen/saliva indicative. Based on the allele-linked DNAm results, p1 could be identified as contributor of semen and p2 as contributor of saliva. Overlapping alleles (length- and sequenced-based) between p1 and p2 are marked (*). Allele-linked (exclusively p1 or p2) DNAm levels (non-)indicative for semen or saliva were labeled as ‘inconclusive’

The FDSTools output of the mixture displays the STR alleles of both contributors, as presented exemplary for D8S1179 (p1: 11, 14; p2: 10, 12) (Fig. 3a). Non-methylated CpG sites at D8S1179-1 and D8S1179-45 were mainly observed on the same reads as allele 11 and 14 (p1). D8S1179 allele 11 (p1) demonstrates semen-characteristic lower DNAm in D8S1179-45 (36.3%) (Fig. 3a, Table 4). Allele 14 of p1 shows higher DNAm (83.9%) and potential overlap to other body fluids which stands in accordance with the observed dependence of repeat-number and DNAm in semen (cf. Fig. 2 and Table S6). If semen DNA was contributed by p2, we would expect lower DNAm levels linked to alleles 10 and 12 as well. For FGA, we observed semen-characteristic lower DNAm in FGA-67 (71.4%) linked to FGA allele 22 (unique p1), while FGA allele 19 (unique p2) exhibits DNA hypermethylation (95.0%), as observed in non-semen samples (cf. Fig. 2, Table 4). All four SE33 alleles show high SE33-73 allele-linked DNAm (>90.8%), which is in concordance with observed high methylation in few semen and all saliva samples (Fig. 2) and can thus not be used for indicative semen/non-semen statements. Furthermore we observed lower DNAm in D1S1677+1 (76.4%) and D19S433-30 (67.4%) linked to allele 18 and 15, respectively, indicating p2 as the non-semen donor.

The FDSTools output for cg21189537 shows that reads containing the alternative allele A in rs79471 (44159148G>A) primarily contain methylated cytosines (CCC), while reads containing the reference allele G contain both methylated and non-methylated cytosines (CCC and TTT) (Fig. 3b). The A-allele-specific DNAm in cg21189537 exhibits hypermethylation levels (99.6%), which is indicative of non-saliva (Table 4, cf. Table 3). This hypermethylation could be linked to p1 (genotype G/A), as p2 has the G/G genotype at rs79471. Contributor p1 has the A/A genotype at rs1078979 neighboring cg20162146, while p2 has the genotype A/G. DNAm linked to the G allele at cg20162146 (p2) exhibits hypermethylation (98.8%) indicative of non-semen. At rs17356301, which is neighboring cg21597595, contributor p1 has a T/T genotype and contributor p2 has a C/C genotype. In the mixture, DNAm linked to the C allele at cg21597595 exhibits an intermediate DNAm indicative of saliva (41.6%), which could be linked to p2. Low DNAm associated with the T allele (p1) indicates non-saliva contribution.

In summary allele-linked DNAm levels in D8S1179-45, FGA-67, D1S1677+1, D19S433-30, cg20162146, cg21597595 and cg21189537 correctly assigned p1 as semen and p2 as saliva contributor in the body fluid mixture. STR-neighboring DNAm and tDMP-iSNP sites are promising targets for body fluid and contributor assignment in mixtures (especially if containing semen), when the alleles of the contributors and the body fluids in question are known. The combined analysis of multiple STRs and iSNPs allows additional support of body fluid identification or exclusion (e.g. saliva or non-saliva). Further experiments including various mixture types as well as unbalanced and low minor component mixtures are needed to evaluate the full potential and limitations. Additionally, decision making approaches for final contributor assignment have to be elaborated.

## Conclusion and outlook

In this study, we successfully analyzed and named the alleles of 18 STRs and 10 iSNPs in bisulfite-converted DNA. As presented, certain challenges resulting from the C>T change during bisulfite conversion must be considered in data analysis. We generated a library file adapted for bisulfite-converted DNA and used FDSTools packages for simultaneous STR/iSNP and DNAm analysis. This offers the opportunity for allele-specific DNAm analysis. We identified novel STR-neighboring DNAm sites that showed differential DNAm levels in forensically relevant body fluids. STR-neighboring DNAm sites alone are not sufficient for determining body fluids. However, some STR systems are potential targets for identifying semen contributors in semen-containing mixtures. Furthermore, we have confirmed the body-fluid-specific DNAm levels of eight tDMPs with neighboring iSNPs as targets for saliva, blood, vaginal fluid and semen contributor assignment. Further research could reveal if DNAm variation around other STRs or in other biological material including organs is present.

Subsequent research will concentrate on the refinement of FDSTools performance and mixture analysis. For instance, DNAm analysis might become an official FDSTools and STRNaming feature, thereby eliminating the necessity for a custom library file. In addition, further marker characterization is required to investigate whether STR alleles, the cell type composition of specific body fluids and chronological age affect levels of DNAm. Suitable markers around STRs will be selected for the purpose of contributor assignment in pre-determined body fluids and combined with tDMP-iSNP targets in a future assay. Input of low-template and degraded DNA material will be tested, as bisulfite conversion negatively affects DNA and the amplification of repetitive sequences can be challenging.

Despite these challenges, our study presents a pilot concept including MPS and the FDSTools software package for simultaneous STR/iSNP and linked-DNAm analysis to identify and link body fluids to mixed-stain contributors and provides foundational methodology that can potentially be used for other DNAm-based applications.

## Supplementary Information

ESM 1 (PDF 285 kb)

ESM 2 (TXT 1 kb)

ESM 3 (PY 8 kb)

ESM 4 (INI 9 kb)

ESM 5 (XLSX 48 kb)

## Declarations

### Clinical trial number

DRKS00030299 (registration date 31.05.23)
