# Supplementary material for: Linking STRs/SNPs and DNA methylation using massively parallel sequencing for potential forensic applications
Source: Int J Legal Med. 2025 Oct 21;140(2):621–37. doi: 10.1007/s00414-025-03602-2 (PMC12956943; doi:10.1007/s00414-025-03602-2)
Supplement: Supplementary file 1 — (PDF 285 kb) [file 414_2025_3602_MOESM1_ESM.pdf]

Linking STRs/SNPs and DNA methylation using massively parallel sequencing for potential forensic applications

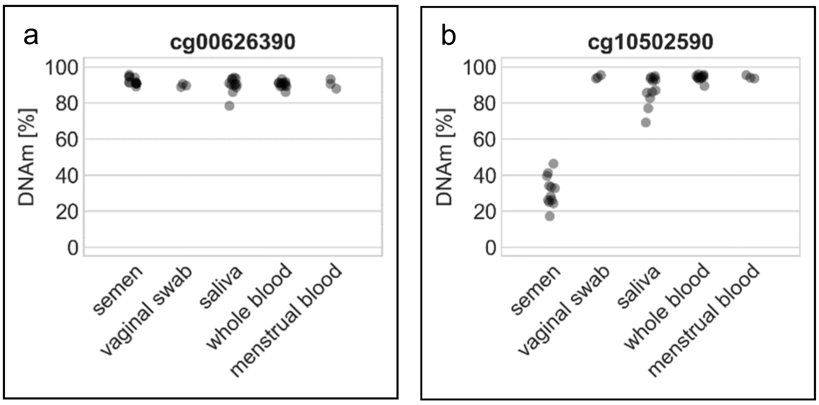

Fig.S1 Individual DNAm values of STR neighboring CpG sites in different body fluids from Infinium Methylation450K BeadChip array data (GSE59509)
Individual DNAm of TPOX neighboring cg00626390 (a) and SE33 neighboring cg10502590 (b) obtained from Infinium Methylation450K BeadChip array data (Gene Expression Omnibus repository dataset GSE59509) in semen (n=12), vaginal swab (n=3), saliva (n=12), whole blood (cg00626390 n=12, cg10502590 n=11) and menstrual blood (n=3). Cg10502590 shows DNA hypomethylation in semen samples.

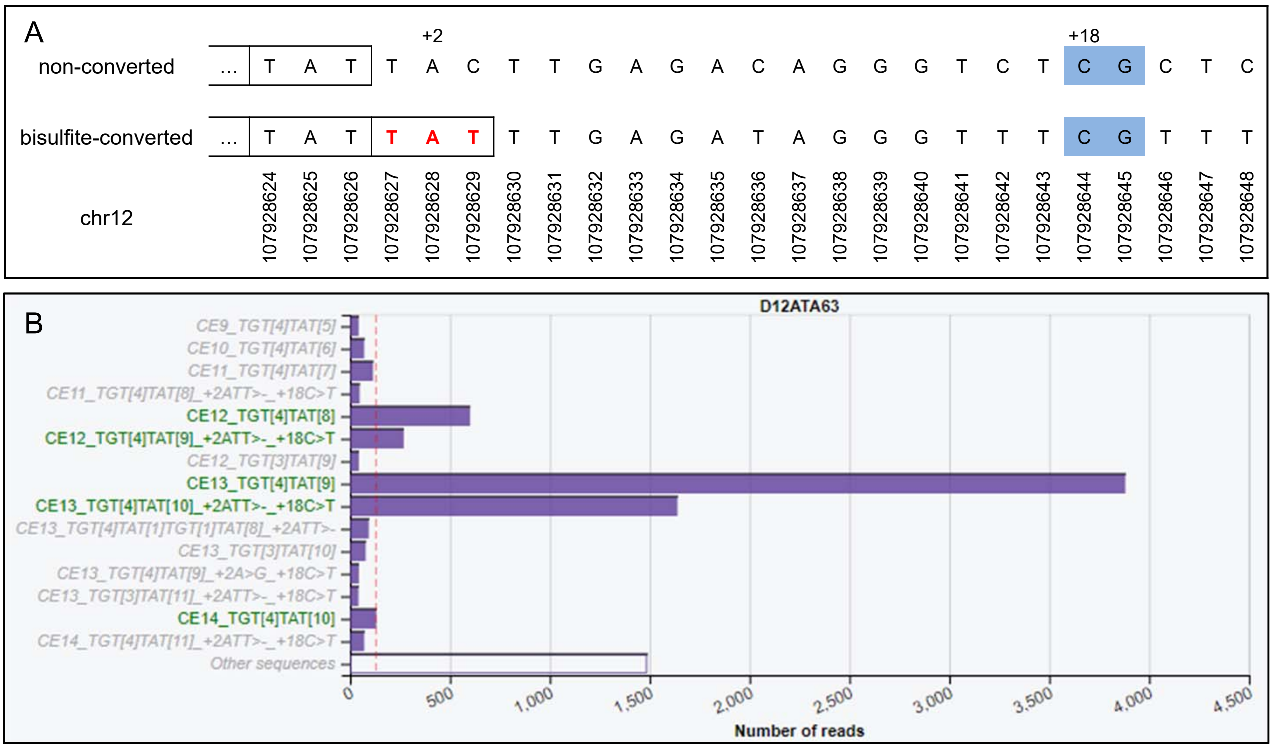

Fig.S2 D12ATA63+18 non-methylation causes allele naming issues
A) 3'-flanking sequence and STRNaming bracketing of D12ATA63 in non-converted and bisulfite-converted DNA. Bisulfite conversion of the TAC bases downstream of D12ATA63 generates an additional TAT repeat (red) in the analyzed sequence. B) Number of D12ATA63 reads of control DNA 9947A named by FDSTools. If +18C>T variant (+18 CpG non-DNA methylation) occurs, difficulties arise in suffix recognition and D12ATA63 boundary detection, resulting in STR structure compensation by +2ATT>-. This is due to an extra, bisulfite-caused TAT repeat at 3'-STR boundary and occurs in genuine alleles (CE13), as well as stutters.

Linking STRs/SNPs and DNA methylation using massively parallel sequencing for potential forensic applications

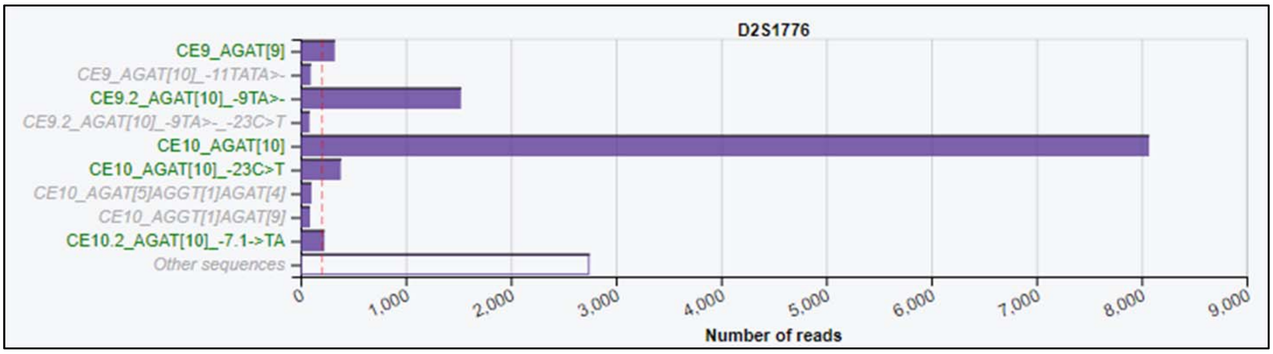

Fig.S3 Analysis of D2S1776 causes multiple stutter artefacts
Number of D2S1776 reads of control DNA 9947A named by FDSTools. Analysis shows minus 4 bp (one repeat, CE9), minus 2 bp (CE9.2), and plus 2 bp (CE10.2) stutter artefacts of reference allele CE10. Stutters consisting of two base pairs are presumably resulting from AT[5] repeat, 14 base pairs upstream of the STR bracketing.

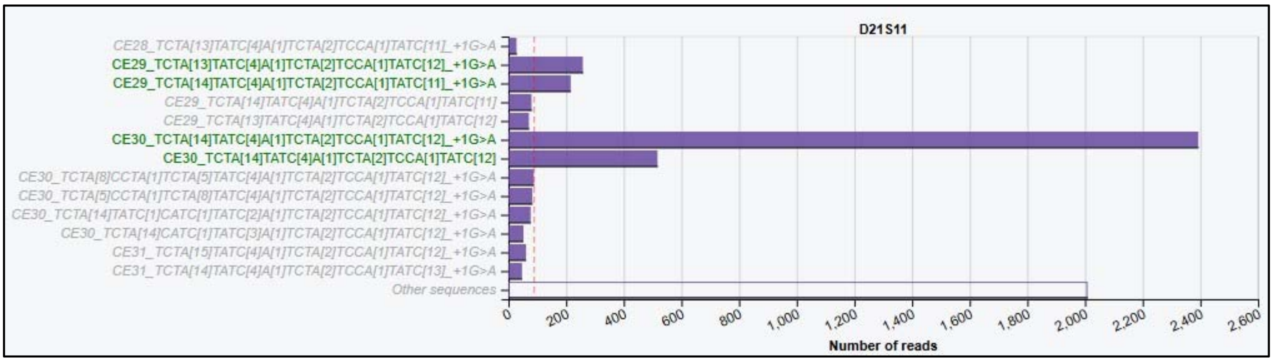

Fig.S4 Stutter artefacts in D21S11 of control DNA 9947A
Number of D21S11 reads of control DNA 9947A named by FDSTools. CE29 (stutter of CE30) consists of two different stutters based on sequence. Allele 30 consists of two long, interrupted repeats (**TCTA[14]**TATC[4]A[1]TCTA[2]TCCA[1]**TATC[12]**) that form individual stutters.
